# Emergence of Nano-Based Formulations for Effective Delivery of Flavonoids against Topical Infectious Disorders

**DOI:** 10.3390/gels9080671

**Published:** 2023-08-18

**Authors:** Khusbu Dwivedi, Ashok Kumar Mandal, Obaid Afzal, Abdulmalik Saleh Alfawaz Altamimi, Ankit Sahoo, Manal A. Alossaimi, Waleed H. Almalki, Abdulaziz Alzahrani, Md. Abul Barkat, Tahani M. Almeleebia, Shehla Nasar Mir Najib Ullah, Mahfoozur Rahman

**Affiliations:** 1 Department of Pharmaceutics, Sambhunath Institute of Pharmacy Jhalwa, Prayagraj 211015, Uttar Pradesh, India; khusbudwivedi99@gmail.com; 2 Department of Pharmacology, Faculty of Medicine, University Malaya, Kuala Lumpur 50603, Malaysia; aakash.am29@gmail.com; 3 Department of Pharmaceutical Chemistry, College of Pharmacy, Prince Sattam Bin Abdulaziz University, Alkharj 11942, Saudi Arabia; obaid263@gmail.com (O.A.); as.altamimi@psau.edu.sa (A.S.A.A.); m.alossaimi@psau.edu.sa (M.A.A.); 4 Department of Pharmaceutical Sciences, Shalom Institute of Health & Allied Sciences, Sam Higginbottom University of Agriculture, Technology & Sciences, Allahabad 211007, Uttar Pradesh, India; ankitsahoo71@gmail.com; 5 Department of Pharmacology and Toxicology, College of Pharmacy, Umm Al-Qura University, Makkah 21955, Saudi Arabia; whmalki@uqu.edu.sa; 6 Pharmaceuticals Chemistry Department, Faculty of Clinical Pharmacy, Al-Baha University, Alaqiq 65779, Saudi Arabia; alzahraniaar@bu.edu.sa; 7 Department of Pharmaceutics, College of Pharmacy, University of Hafr Al Batin, Al-Batin 39524, Saudi Arabia; abulbarkat05@gmail.com; 8 Department of Clinical Pharmacy, College of Pharmacy, King Khalid University, Abha 61421, Saudi Arabia; talmelby@kku.edu.sa; 9 Department of Pharmacognosy, Faculty of Pharmacy, King Khalid University, Abha 62529, Saudi Arabia

**Keywords:** flavonoids, nanomedicine, topical infection, encapsulation, drug delivery systems, nanogel, hydrogels

## Abstract

Flavonoids are hydroxylated phenolic substances in vegetables, fruits, flowers, seeds, wine, tea, nuts, propolis, and honey. They belong to a versatile category of natural polyphenolic compounds. Their biological function depends on various factors such as their chemical structure, degree of hydroxylation, degree of polymerization conjugation, and substitutions. Flavonoids have gained considerable attention among researchers, as they show a wide range of pharmacological activities, including coronary heart disease prevention, antioxidative, hepatoprotective, anti-inflammatory, free-radical scavenging, anticancer, and anti-atherosclerotic activities. Plants synthesize flavonoid compounds in response to pathogen attacks, and these compounds exhibit potent antimicrobial (antibacterial, antifungal, and antiviral) activity against a wide range of pathogenic microorganisms. However, certain antibacterial flavonoids have the ability to selectively target the cell wall of bacteria and inhibit virulence factors, including biofilm formation. Moreover, some flavonoids are known to reverse antibiotic resistance and enhance the efficacy of existing antibiotic drugs. However, due to their poor solubility in water, flavonoids have limited oral bioavailability. They are quickly metabolized in the gastrointestinal region, which limits their ability to prevent and treat various disorders. The integration of flavonoids into nanomedicine constitutes a viable strategy for achieving efficient cutaneous delivery owing to their favorable encapsulation capacity and diminished toxicity. The utilization of nanoparticles or nanoformulations facilitates drug delivery by targeting the drug to the specific site of action and exhibits excellent physicochemical stability.

## 1. Introduction

The human body needs protection against harmful substances and microbes that may enter it. As the primary defense, the skin protects the body against different chemical and microbial agents, protects the bones, muscles, and blood vessels, and serves against various abrasions. Aside from the main skin diseases and injuries, there are many other factors and underlying diseases that can cause skin infections. These include diabetes mellitus, the use of steroids (clobetasol propionate, fluocinonide, betamethasone dipropionate, and fluticasone propionate) topically or systemically, and immune deficiency diseases. Various microbes such as viruses, bacteria, fungi, or parasites reside on the skin and become a significant cause of skin infections if they penetrate the skin’s defenses. Common types of infectious agents that are involved in causing infection in the skin and soft tissues include *Clostridium per-fringes*, *Streptococcus pyrogens* (Group A hemolytic *streptococcus*), *Staphylococcus aureus*, and the *bacteriodes* group, *Pasturella tulurensis*, *Neisseria gonorrhea*, *Bacillus antracis*, *Mycobacterium tuberculosis*, *Mycobacterium leprae*, and *Pseudomonas aeruginosa*. The common fungi involved in skin infections are *Candida albicans*, *Epidermophyton flocossum*, *Candida neoformans*, *Trichophyton tonsurans*, and *Melassezia furfur*. Skin infections include two categories, i.e., primary and secondary infections [1]. Primary infections are those in which normal skin is affected by single pathogens. Pathogens like coryneform bacteria, β hemolytic streptococci, and *S. aureus* are the common causes of prion diseases. 

The clinical picture and course of infection vary according to the underlying disease condition. Common primary skin infections include folliculitis, boils, and impetigo. Secondary infections develop in already diseased skin. Intertrigo and toe web infections are examples of secondary infections. Oedema, erythema, or other signs of inflammation and sometimes a local accumulation of fluid (vesicles, bullae) or pus (furuncles) are commonly observed in topical infectious disorders. The clinical expression and pattern of an infectious disease are influenced by the layers of skin, the underlying disease, and the disease-causing bacteria.

Conventional drugs used to treat topical infectious diseases are a practical approach, but conventional drugs can cause adverse effects such as skin irritation, redness, itching, staining, and skin cancer. As a result, herbal medicines are becoming an increasingly popular alternative treatment for skin infections. Treatment with plant-obtained natural drugs has gained popularity, as they have several advantages, including better patient compliance, being less expensive, and having fewer side effects with easy acceptance [2]. The use of medicinal plants containing flavonoids has been a part of traditional medicine across the globe to treat skin diseases. When microbes infect plants, they synthesize hydroxylated phenolic compounds in response to microbial infection, which are flavonoids. The pharmacological benefits of flavonoids and their potential as supplementary bioactive components to mitigate human disease have led to a surge in research exploring flavonoid compounds as newer antibiotic therapies [3]. It has been demonstrated that flavonoids have several health-promoting physiological properties. The antimicrobial properties of flavonoid-rich natural products have been studied and recorded in the scientific literature for many years. *Acalypha wilkesiana* is a topical shrub commonly used as an ornamental plant in many parts of the world, including southern Nigeria. In traditional medicine, *Acalypha wilkesiana* has been used as an herbal remedy for various conditions, including skin infections in children [4].

Argentinean folk used Tagetes minuta, which contains quercetagetin-7-arabinzylgalactoside, to treat infectious diseases associated with the pathogens *B. subtilis*, *S. epidermidis*, *S. aureus*, *E. coli*, and *P. aeruginosa* [5]. Tripleurospermum disciform, also known as mayweed, has a richness of flavonoids, including luteolin, apigenin, quercetin, and kaempferol as their glycosides. According to studies by Benjamen et al. [6], leaf juice and decoctions of *Senna alata* have been used to treat ringworm and other skin diseases. Several herbs traditionally employed for skin infection management include *Ramunculus scleratus*, *Quisqualis indica*, *Amaranthus spinosus*, *Cassia alata*, and *Cormelina benghalensis Retama raetam* flower extract. As shown in Table 1, flavonoids like apigenin, galanin, flavone and flavanol glycosides, isoflavones, flavanones, and chalcones have a lot of antibacterial activity [7].

Pharmaceutical companies face numerous challenges in the delivery of phytoconstituents. However, these challenges can be addressed by incorporating phytoconstituents into nanoformulations. Nanoformulations (such as liposomes, polymeric nanoparticles, solid lipid nanoparticles, and transferosomes) offer several advantages, including improved solubility, stability, distribution in tissues, sustained delivery, and protection from degradation, which aid in the improvement of dosage form and provide numerous benefits for herbal constituents. These nanocarriers can be further incorporated into gel to improve patient conditions. For example, Luo et al. [8] formulated a biocompatible gliadin-sericin complex colloidal particle for topical delivery of phloretin, which overcomes the various hurdles associated with the solubility and stability of phloretin and improves its topical delivery, antioxidant property, and cell uptake-capacity. Another study by Sadeghi-Ghadi et al. [9] investigated the utilization of nanovesicles for encapsulating curcumin and quercetin, both with and without the inclusion of hyaluronan. The utilization of nanovesicles has been found to enhance the effectiveness of topical drug delivery systems.

**Table 1:** Flavonoids containing antimicrobial activity and general mechanism of action.

Antimicrobial Activity against	Flavonoid Examples	General Mechanism of Action	References
Fungi	7-hydroxy-3,4-(methylenedioxy) flavan, 6,7,4-trihydroxy-3-5-dimethoxyflavon, 5,5-dihydroxy-8,2,4-trimethoxyflavone, 5,7,4-trihydroxy-3,5-dimethoxyflavone	Induced plasma membrane disruption, inhibition of cell wall formation, mitochondrial dysfunction, inhibition of cell division, inhibition of efflux pumps, inhibition of RNA/DNA, and protein synthesis	[10,11]
Bacteria	Apigenin, Galangin, Genkwanin, Pinocembrin, Naringin and Naringenin, Epigallocatechin gallate, Luteolin and Luteolin 7-glucoside, Quercetin, 3-O-methylquercetin, Kaempferol	Membrane disruption, biofilm formation, cell envelope synthesis inhibition, electron transport chain, and adenosine triphosphate (ATP) synthesis	[11,12]
Viruses	Baicalein, Robustaflavon, Hinokiflavon, Demethylated gardenin A, Robinetin, Myricetin, Baicalein, 3,2-dihydroxy flavon, Rutin, Pelargonidin, Leucocyanidin,	Blocked attachment and entry of the virus into cells, interfered with various stages of viral replication processes or translation and polyprotein processing to prevent the release of the viruses to infect other cells	[11,13]

This review will provide an overview of flavonoids and explore how nanoencapsulation methods can improve their therapeutic effects. At the end of the review, we discuss examples of nanoformulation-loaded flavonoids that have been developed.

## 2. Bioavailability and Toxicity of Flavonoids

One of the most significant issues with using flavonoids is their limited oral absorption. Even with a high intake of flavonoids, plasma and tissue concentrations are frequently inadequate to deliver the intended pharmacological effects. However, certain limitations are associated with the delivery of flavonoids, including their molecular weight, chemical structure, and lipophilic nature, resulting in a reduced capacity for oral absorption. Their property of high first-pass metabolism resulting in quick elimination has reduced their bioavailability. In addition, complexation, or precipitation upon administration with other food components and microbiota degradation, changes in pH, temperature change, U.V. radiation, and exposure to oxygen may reduce stability and bioavailability [14]. The wide distribution of flavonoids in edible plants and beverages and their use in traditional medicine have led to the assumption that they have low toxicity. However, flavonoids exhibit diverse activities in mammalian cells, and in vivo testing would be required to fully evaluate their practical usefulness in modern medicine and determine their potential side effects [15], given that the selectivity of flavonoids for eukaryotic enzymes varies among different compounds.

So, putting flavonoids into an effective nanocarrier system can improve their pharmacokinetic and therapeutic potential and keep them from being harmful or having side effects. As they can interact with the metabolism of drugs, and given their fast metabolic elimination, the need to develop a novel way to improve the delivery of flavonoids should be emphasized [16]. Topical delivery of active drug molecules is one of the most popular approaches to increasing patient compliance. Cutaneous application emerges as an alternative solution for prevalent oral and parental problems.

## 3. Mechanism of Action of Flavonoids in Topical Infections

**Figure 1:**
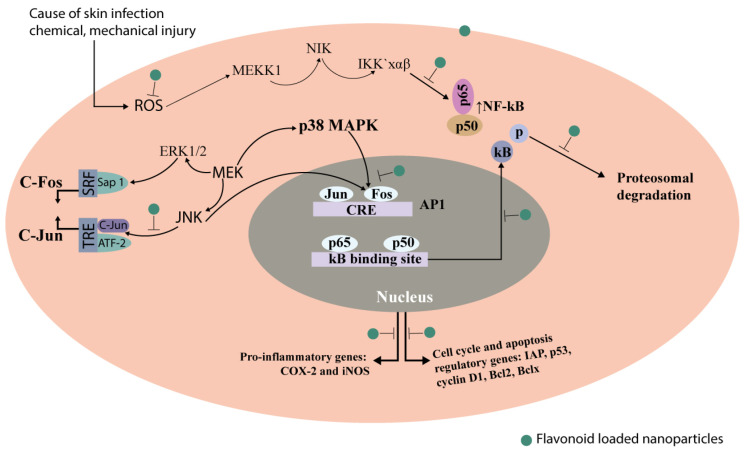
The activation of different cell-signaling pathways in the skin layer by forming reactive oxygen species (ROS). Several signaling pathways are triggered due to chemical and mechanical injury-mediated ROS generation during the pathogenesis of different skin disorders. ROS are responsible for stimulating mitogen-activated protein kinases (MAPKs), the most significant of which are the extracellular regulated kinase (ERK), JNK, and p38 kinases. ERK and JNK are required to recruit c-Fos and c-Jun to the nucleus, where they activate the transcription factor AP-1, whereas p38 and inhibitory kappa kinases (IKK) are required for the transcriptional stimulation of NF-κB. Both of these factors are essential in controlling a wide range of genes involved in the pathogenesis of inflammation (such as ins and COX-2) and in regulating cell cycle, proliferation, and apoptosis. (Cyclin D1, Bcl2, Bclx, IAP, p21).

The antioxidant and anti-inflammatory properties of flavonoids have been investigated, making them useful in treating topical diseases. The precise mode of action of flavonoids in topical conditions is concealed. However, it is believed to involve integrating several mechanisms, including antioxidant activity, anti-inflammatory activity, and signaling pathway modulation. Flavonoids have already been shown to be responsible for radical scavenging activity by the ease with which they donate hydrogen atoms to active free radicals [17]. Also, they have indirect antioxidant effects by making their own protective enzymes and by regulating signaling pathways in a good way. Because inflammation is one of the main driving factors of skin pathology and a crucial component in the formation and spread of many skin diseases, diminishing it can help alleviate symptoms and promote recovery. The role of cytokines in inflammatory events is well known, and their signaling pathways are significant prospects for therapeutic action in the search for possible vesicant injury countermeasures. As shown in Figure 1, there are several Janus kinase (Jak)-signal transducers and activators of transcription (Stat) networks [18] that help cytokines talk to each other. Flavonoids have been demonstrated to alter several cellular communication pathways, which may impact the emergence of skin disorders. They can control the action of enzymes involved in the etiology of topical illnesses as well as the production of genes involved in oxidative stress and inflammation. Overall, flavonoids have the potential to benefit the therapy of topical diseases due to their antioxidant and anti-inflammatory qualities, as well as their ability to regulate cellular signaling pathways. However, more investigation is required to completely comprehend the precise processes of action and the ideal dosage and formulation for use in humans.

## 4. Why Nanoencapsulation Is Necessary for Cutaneous Flavonoid Delivery

**Figure 2:**
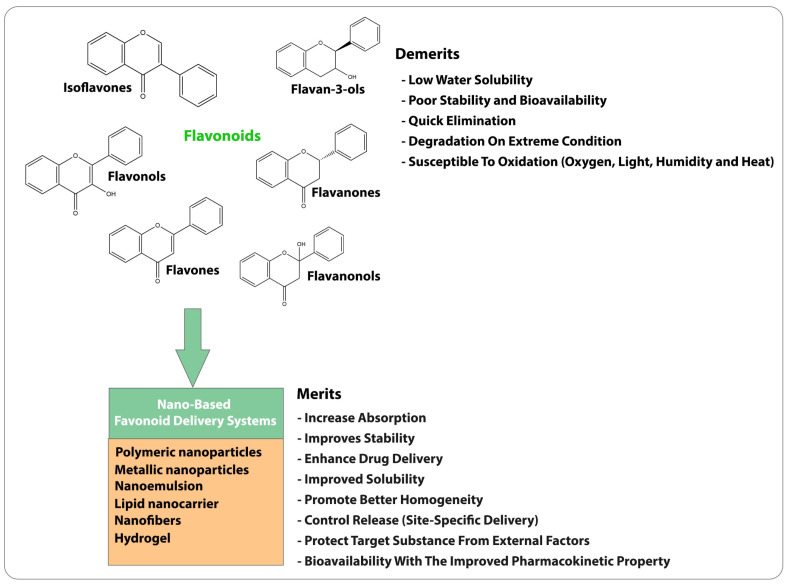
Conventional delivery versus nanoencapsulation approaches to enhance their applicability.

Administering flavonoids through the skin is a potential alternative delivery method. This topical administration offers advantages such as the controlled release of the drug and protection from degradation by gastric and intestinal fluids. However, the stratum corneum presents a significant obstacle to skin penetration of the flavonoids, as shown in Figure 2. This challenge can be overcome by the nanoencapsulation approach for delivering flavonoids, as it can overcome several limitations associated with the conventional delivery method. Nano based delivery systems such as liposomes, hydrogels, nanofibers, solid lipid nanoparticles, and polymeric micelles are some of the effective delivery systems that give site-specific delivery, improve the solubility profile, improve bioavailability, and protect flavonoids from degradation. Additionally, nanoencapsulation can enhance flavonoid activity by improving cellular uptake and reducing toxicity. Further, these delivery systems can be incorporated into topical formulations like creams, gels, and ointments for patient compliance. Nanoformulation protects these flavonoids from degradation. Therefore, nanoencapsulation holds excellent potential for improving the therapeutic efficacy of flavonoids and expanding their clinical applications [14].

### Nanomedicine: Merits and Demerits

By delivering certain compounds to the skin, novel drug delivery systems can make them more effective and safer even though they have poor physical, chemical, and pharmacokinetic properties. Even though significant advances have been made in delivery systems, developing a viable and effective system remains a challenging task. Careful selection of a vehicle compatible with the active agent is crucial for success. Moreover, the safety of the selected molecules, potential harmful degradation products, and the final product’s high cost pose significant limitations that must be overcome [15]. Nanocarriers have been shown to enhance the properties of drugs by improving their blood circulation half-life, diffusivity, solubility, and immunogenicity. However, developing a successfully targeted delivery vehicle relies on specific prerequisites, such as the physiochemical and biological properties of the carrier. Surface hydrophilicity and surface charge can affect particles’ circulating half-life [19]. Nanoparticles loaded with small molecules, peptides, or nucleic acids are less likely to be recognized by the immune system. Targeting ligands can also improve cellular absorption via receptor-mediated endocytosis. Despite these benefits, limitations still exist in using nanocarriers, including storage, unexpected pro-inflammatory responses, and the generation of pro-oxidation chemical species, which must be considered during the design process. Hydrogels are one of the most intriguing carrier systems in the medical sciences. Hydrogels’ adaptability to incorporating hydrophilic and hydrophobic drugs makes them versatile for incorporating flavonoids, thereby increasing their applicability in drug delivery systems. They are widely used in topical drug delivery systems. Hydrogels improve drugs’ penetration ability in target tissues, increasing efficacy [20]. Hydrogels improve the bioavailability of flavonoids, ensuring greater reach of the active ingredients to the targeted site. Hydrogels can release the drug slowly for a prolonged duration, optimizing therapeutic effects and minimizing fluctuations in the concentration of the drug. These advantages help develop more effective and targeted therapies for various skin diseases.

## 5. How Do Nanocarriers Work to Combat Topical Infectious Disorders

**Figure 3:**
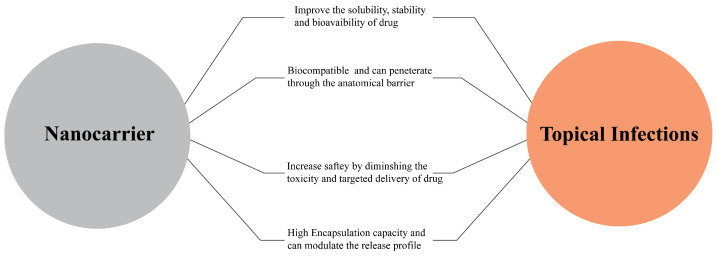
Lucrative merits of nanocarriers in combating topical infections.

The prevalence of topical infection disorders has been a notable burden [21] in the scenario of pharmacotherapy curing them with minimal side effects for the tissue. The lucrative merits of dermal drug delivery cargo, such as lower toxicity and side effects, elevated patient compliance, high bioavailability, low cost, and quick medication discharge, have attracted scientists to explore novel drug discovery and delivery of these drugs [22]. The foremost challenges with dermal drug delivery are penetrating the stratum corneum of the skin epidermis, which impairs the efficacy of drugs [23]. However, the rise of nanocarriers has revolutionized topical drug delivery. A plethora of nanoscaled cargoes like lipid nanocarriers, polymer-based nanoparticles, and inorganic nanoparticles have gained much consideration in drug delivery in topical infectious disorders. Nanostructured carriers in pharmaceutical science are the most recent and advanced approach to delivering lead compounds of various molecular weights to targets. It offers targeted drug delivery and can be used as the protective casing for modulation of the release profile, minimizing toxicity and adhesivity to the skin (as shown in Figure 3). The physiochemical characteristics of nanocarriers, such as size and charge, rigidity, and hydrophobicity, favor the convenient delivery of drugs to skin layers to combat the challenges of drug delivery in topical infectious diseases. Additionally, the nano-size of the carrier increases the specificity of drugs, minimizing the toxicity and dose of the drug to be administered, with the potential to penetrate through several anatomical barriers [24,25]. Lipid nanocarriers have clinched more interest due to their biocompatibility and lower toxicity. The lipid nanocarrier’s resemblance to the skin’s fatty acids amplifies the drug’s penetration. Specifically, polymer-based nanoparticles are one of the best candidates for drug delivery for topical disorders. They are structurally stable and can maintain their structure for an extended time when topically applied. Conceptually, the polymeric nanocarrier’s lipidic core is surrounded by an ultrathin polymeric wall stabilized by surfactant, and the diffusion of active pharmaceutical ingredients from the core can be controlled using the polymeric wall’s different characteristics [26,27]. Interestingly, the thermosensitive drugs can also be encapsulated below critical temperature so they can be released at the inflamed site on the skin (like psoriasis), where the temperature is likely elevated. Thus, smart drug delivery of drugs can be achieved by stimulus variation on the skin [28]. Similarly, the application of inorganic nanoparticles offers distinct advantages in terms of precise targeting and regulation of their cellular activities. Inorganic nanoparticles exhibit remarkable stability over extended durations and diverse properties, making them highly suitable for targeted drug delivery [29].

## 6. Nanocarriers in Their Effective Delivery against Topical Infectious Disorders

### 6.1. Lipid-Based Nanoparticles

Lipid-based nanoparticles are the most extensively studied type for topical use, focusing on liposomes, solid nanoparticles, nanoemulsions, and nanostructured lipid carriers as the primary types of matrix nanoparticles. In contrast, elastic liposomes are the primary type of vesicular particles analyzed in permeation studies.

#### 6.1.1. Elastic Liposomes

Elastic liposomes are a novel concept that enables conventional liposomes to deform and flow through narrow pores in the skin, such as capillaries [30]. These deformable fluid vesicles have been shown to enhance skin permeation and the delivery of active compounds to deeper layers of the skin. Lipid-based nanoparticles have been studied a lot as production, characterization, and application delivery systems because they have useful properties like occluding, increasing hydration, having different release profiles, and better skin penetration with targeted effects. A study by Kianvash et al. [5] prepared a liposome containing curcumin-propylene glycol for a dermal delivery system to treat burn wounds. In vitro release studies show a release of 50% and 60% of loaded curcumin after 7 and 24 h, respectively, indicating an extended-release period compared to free curcumin dispersion, i.e., 96% in 24 h. Gram-positive bacteria, including *E. faecalis*, *S. aureus*, *B. subtilis*, *B. cereus*, and *S. epidermidis*, and *P. aeruginosa* as the gram-negative bacteria, were detected on rats’ wounds. The antibacterial study reveals the mean ± S.D. to be 6.9 ± 0.23 × 10^4^ CFU/mL in 4 days and 8 ± 0.13 × 10^3^ CFU/mL in 8 days, which implies a 98.8% colonization reduction. Irina I. et al. [30] demonstrated the antimicrobial effect of *Rosmarinus officinalis* L., a loaded liposome. The liposomes formed had suitable particle sizes and high encapsulation efficiency. Antimicrobial activity against MSSA, MRSA, *B. cereus*, *E. faecalis*, and *E. coli* was reported. Madan et al. investigated the effect of a liposomal gel containing curcumin and lauric acid in a rat ear model against *P. acnes*-induced infection. The combination of curcumin and lauric acid in a ratio of 1:1 in the liposomal gel showed a significant elevation in the antibacterial effect against both macrolide-resistant and macrolide-sensitive strains of *P. acnes* [31]. Chen et al. [32] formulated an (+)-catechin-loaded liposome for topical application, and three types of liposomes were compared. They were the most effective for catechin delivery to the skin, with a higher aqueous volume and a greater entrapment efficiency of 50.0 ± 5.9%. All liposomal formulations displayed prolonged catechin release. However, the deformable resveratrol (REV) liposomes demonstrated significantly better catechin deposition than the other formulations or catechin solutions. Joraholmen et al. [33] developed a REV-loaded liposome incorporated in chitosan hydrogel for the localized treatment of bacterial (*Chlamydia trachomatis*) infection, the most common cause of sexually transmitted disease (STD). The deformable REV liposomes had particle sizes ranging from 335.6 to 551.1 nm. Their studies found that free REV and REV-liposome-in-chitosan hydrogel inhibited propagation in a concentration-dependent manner. However, the REV-liposome-in-chitosan hydrogel system showed inhibition of 94% and 78% for 3 and 1.5 µg·mL^−1^, respectively, compared to 72% and 43% for free REV, respectively. Therefore, in the end, it was concluded that liposomes and elastic liposomes were demonstrated to be effective topical drug delivery carriers for the delivery of flavonoids.

#### 6.1.2. Nanostructured Lipid Carriers

To address the limitations of SLNs, a new generation of lipid nanoparticles known as nanostructured lipid carriers (NLCs) has emerged. NLCs are made with lipids (both solid and liquid) and emulsifiers that are biodegradable and compatible. To prevent drug leakage and provide a high drug load, incorporating liquid lipids (oil) causes structural imperfections in solid lipids, leading to a less ordered crystalline arrangement. As an alternative to SLNs, polymeric nanoparticles, emulsions, microparticles, liposomes, etc., NLCs have attracted the attention of researchers in recent years [34]. Hydrophilic and lipophilic drugs can both be transported effectively by these nanocarriers. NLCs are a novel and potentially useful carrier system for the transdermal, topical, pulmonary, ocular, and oral administration of drugs. NLCs have recently found applications in the drug delivery of cosmeceuticals, nutraceuticals, and phytoconstituents against various disorders including topical disease [34]. Barros et al. [34] evaluate the synergistic effect of quercetin with natural plant oil-based NLCs (NPO-NLCs). In their study, it was found that blank NPO-NLCs also exhibit antimicrobial activity. When compared, quercetin-loaded NPO-NLCs showed the highest antimicrobial activity against *S. aureus* compared to blank NPO-NLCs. It was concluded that by incorporating quercetin, the concentration required to inhibit the growth of *S. aureus* decreased from 6.25 to 0.16 mg·mL^−1^, indicating its potential for treating skin infections. Elkhateeb et al. [35] investigated the effectiveness of propolis NLCs for their wound-healing, antimicrobial, and antifungal properties. They prepared the NLCs and compared them to raw propolis extract (EXTR). The study found that propolis-NLCs had significantly elevated flavonoid and phenolic contents compared to propolis-EXTR (two-fold and nine-fold increases, respectively). The Propolis-NLCs showed a two-fold higher inhibitory effect on various microbes, including gram-positive, gram-negative, and *C. albican*, compared to Propolis-EXTR. The study concluded that propolis-NLCs have a broad-spectrum antibacterial effect and higher skin regenerative potency than propolis-EXTR. Therefore, the use of nanotechnology also impacts the herbal extract, leading to increased flavonoid content, improved antioxidant and antimicrobial effects, and suggesting that propolis-NLCs could be a potential therapy for wound healing.

#### 6.1.3. Solid Lipid Nanoparticles

To combat the limitations of traditional colloidal carriers like liposomes and polymeric nanoparticles, SLNs were introduced in the 1990s [36,37]. Because of their high drug-loading capacity, ability to modulate drug release, and protection against physical and chemical degradation, SLNs have attracted a lot of attention in the pharmaceutical sciences for use in drug delivery applications. Solid lipids are dispersed in an aqueous surfactant medium to form nanosized lipid particles known as SLNs [37]. Biodegradable lipids with a high melting point, like triglycerides, partial glycerides, fatty acids, fatty alcohols, and waxes, are typically used in the preparation of SLNs [37]. By lowering the surface tension between water and lipid, surfactants are used to keep the structure of SLNs from collapsing. Kaempferia parviflora (KP) is known to have antimicrobial and anti-inflammatory activity. The extract shows antibacterial activity against *S. epidermidis* with MICs ranging from 3.84 mg·mL^−1^ and Cutibacterium acnes with 0.015 to 0.030 mg·mL^−1^ [36]. Khaetthareeya et al. [37] developed KP-loaded SLNs focused on the transdermal permeability of the extract. The SLNs achieved particle sizes ranging from 82 to 108 nm, with high entrapment efficiencies of up to 87%. They compared the transdermal delivery of KP extract-loaded SLNs to gel formulations containing KP extract with hydroxypropyl methylcellulose gel or Tween 80. Comparing both, the permeability through the skin significantly differed between the SLN formulation (95.57 ± 9.08 g) and the gel formulation (81.04 ± 5.82 g), respectively. Therefore, from this study, it was concluded that SLNs could be a promising option for topical drug delivery.

#### 6.1.4. Nanoemulsions

A nanoemulsion is a type of emulsion that contains tiny droplets, typically between 20 and 500 nanometers in size. It is a clear and stable dispersion system that can be either oil-in-water or water-in-oil. A layer of surfactant molecules stabilizes the interface between the droplets and the surrounding liquid. Nanoemulsions can improve the delivery and efficacy of lipophilic molecules, are more acceptable to patients, increase skin permeation rates, and enhance topical drug effects by residing in the uppermost skin layers for longer periods due to their large surface area and low surface tension [38]. A study was conducted by Bidone et al. [39] on topical nanoemulsions of *Achyrocline satureioides* extract (ASE). A topical nanoemulsion loaded with ASE had more pronounced inhibitory effects on Herpes Simplex Virus type 1 replication than ASE or pure quercetin. A significant reduction in concentration, which inhibits 50% of HSV-1 plaque number, with a decrease in IC50 value from 14.07 to 1.40 µg/mL, was reported. After applying ASE-loaded nanoemulsion topically, the flavonoids (quercetin, luteolin, and 3-O-methyl quercetin) were detected in the upper mucosa layer and skin epidermis, based on their distribution in porcine skin and mucosa. Additionally, more flavonoids were detected in impaired tissues, particularly in deeper mucosal layers. These results suggested that the tested ASE-loaded nanoemulsion could be applied topically to treat herpes infections. This study’s value lies in identifying a potential alternative treatment for herpes infections using a natural extract, which could have fewer side effects than current antiviral drugs. In a study by Zhao et al. [40], the antibacterial activity of a self-nano-emulsifying drug delivery system employing buckwheat flavonoids was assessed. Buckwheat flavonoid nanoemulsion was compared to buckwheat flavonoid suspension for its antibacterial effectiveness against *C. albicans*, *S. aureus*, and *E. coli* [40]. The agar disc diffusion test showed that the suspension’s inhibition zone diameter was about half of the inhibition zone diameter of the nanoemulsion against three bacteria. Therefore, based on the above findings, it was concluded that the nanodroplet size of the nanoemulsion provides greater area, higher diffusion, and higher antimicrobial action.

### 6.2. Polymer-Based Nanoparticles

Polymeric nanoparticles have gained wider attention in topical drug delivery because they enable a controlled release of the active ingredients they enclose. Due to their rigid matrices, these nanoparticles are structurally robust and can preserve their form for an extended period upon topical application. These structures can be classified into two categories depending on their shapes: nanospheres (porous matrixes in which the drug is uniformly dispersed) and nanocapsules (vesicular systems with a drug held in the core and surrounded by a polymeric membrane). Many flavonoid-loaded polymeric nanoparticles have been developed and characterized for their antimicrobial properties. In a study [41], curcumin nanoparticles were developed using the sonication method, and their effectiveness against *S. aureus*, *P. aeruginosa*, and *E. coli* was tested in the lab. These curcumin nanoparticles were incorporated into a cream. The curcumin nanoparticle-incorporated cream was reported to be highly effective against *P. aeruginosa* (with a 30 mm zone of inhibition) and moderately effective against *S. aureus* (with a 20 mm zone of inhibition). The cream containing curcumin nanoparticles showed good epithelization in wound healing. Poly(lactic-co-glycolic acid) (PLGA) nanoparticles were made by Dongdong S et al. [42], who used the emulsion-solvent evaporation method. These nanoparticles were loaded with quercetin and coated with poly(vinyl alcohol) to stay stable. The resulting quercetin-loaded PLGA nanoparticles (QTs-PLGA nanoparticles) had an average size of 100–150 nm and were spherical in shape. Further, they reported 100% entrapment efficiency. They evaluated the antimicrobial activity of QTs-PLGA nanoparticles against different bacterial strains (*B. subtitlis*, *Micrococcus tetragenus*, *S. aureus*, *P. aeruginosa*, and *E. coli*), comparing their effectiveness with free quercetin. The result showed that QTs-PLGA nanoparticles had a significant inhibitory effect on all tested bacterial strains. Among the gram-negative bacteria, *E. coli* was the most susceptible to QTs-PLGA nanoparticles, while *P. aeruginosa* exhibited the next level of susceptibility. Among the gram-positive bacteria, *M. tetragenous* was the most susceptible to QTs-PLGA nanoparticles. The study also indicates that quercetin alone had a stronger inhibitory effect on *E. coli* compared to *M. tetragenus*. This finding suggests that quercetin may impart some selectivity to QTs-PLGA nanoparticles, possibly due to differences in cell membrane constituents and cell wall structures between different bacterial strains. The minimum inhibitory concentration (MIC) for *E. coli* was said to be 5 ± 0.3 µg/mL, while for *M. tetragenus*, it was found to be 12 ± 0.1 µg/mL. Rofeal et al. [43] have developed a functional combination of polyhydroxy butyrate (PHB) and chitosan (Cs) for addressing wound infection. To improve the transdermal delivery of the water-insoluble kaempferol (KPF) through the skin, they converted it into water-soluble KPD nanocrystals (KPF-NCs) with a particle size of 145 ± 11 nm and excellent colloidal stability (−31 ± 0.4 mV). Through their research, they determined that a PHB-Cs-KPF-NC film with a 1:2 ratio displayed the most favorable physical properties, including high stability, thermal endurance, and mechanical strength (33 ± 1 MPa). This film exhibited an exceptional encapsulation efficiency of 96.6% and sustained release for over 48 h. Moreover, the developed film containing a naturally sourced blend of Cs and KPF-NCs demonstrated powerful antibacterial effects against multidrug-resistant *S. aureus* and *Acetobacter baumannii* even at very low concentrations. In vitro testing revealed an improved antibacterial nature of the membrane, resulting in nearly complete inhibition of cell viability against the tested strain after 48 h. So, from all the above studies, it can be concluded that polymeric nanoparticles can be an effective delivery system for treating various topical infectious diseases.

### 6.3. Hydrogels

Hydrogels are gel-like materials made from biocompatible polymers that can absorb and retain large amounts of water while maintaining a 3D structure [44]. The polymer network of hydrogels forms a mesh-like structure that can be customized to trap drugs and control their diffusion and release. Additionally, the size of the pores in the hydrogel network can be adjusted to allow drug molecules to interact with the polymer chain and ensure proper encapsulation [14]. Many flavonoid-based hydrogels have been developed and evaluated for their high cutaneous hydration and drug delivery levels, making them suitable candidates for transdermal delivery. Agrawal et al. [45] developed a hydrogel system loaded with Theobroma cacao extract (TCE) for improved antioxidative and antimicrobial effects. The extract contains phenolic compounds, catechin, procyanidin flavonoids, methylxanthines, and epicatechin phytoconstituents. The optimized hydrogel was homogeneous and free of phase separation, precipitation, or agglomeration. The therapeutic index of the TCE was greatly enhanced by the hydrogel-based version, which improved its suitability for transdermal administration. The study found that the total flavonoid content in TCE was 68.02 ± 0.59 mg/mL, and the cocoa gel demonstrated antibacterial activity against some sensitive bacterial and fungal strains. Specifically, the cocoa gel demonstrated antibacterial activity against *P. fluorescens*, *B. licheniformis*, and *M. leuteus*, and antifungal activity against *R. oryzae*, Trichoderma, and *A. niger*. Archana et al. [46] prepared a hydrogel containing curcumin-loaded cubosomal to enhance curcumin activity in topical drug delivery. The morphological study of the curcumin-loaded cubosomes shows a particle size of 75.2 nm with a zeta potential of −24 mV. The entrapment efficiency of the cubosomal formulation was 86.4%. In the antimicrobial efficacy study, the zone of inhibition for cubosomes was measured at 16.20 ± 4.26 mm, whereas pure curcumin exhibited a zone of inhibition of 11.36 ± 1.14 mm after 24 h. Moreover, the hydrogel containing cubosomes demonstrated a higher zone of inhibition than pure curcumin. In a randomized, double-blind, controlled clinical trial, the anti-acne activity of *Nigella sativa* L. (black cumin, black seed) hydrogel was investigated. It was a study on 60 patients, with half of them (30 patients) receiving an *N. sativa* hydrogel and the other half receiving a placebo hydrogel twice daily for 60 days. The efficacy of the *N. sativa* hydrogel in treating acne was evaluated using the Investigator’s Global Assessment (IGA) grading scale and the Acne Disability Index (ADI) questionnaire. The results showed a significant reduction in IGA score for the patient group, with an impressive mean reduction of 76%. In comparison, the placebo group had a minimal mean reduction of 3.3% [47]. Additionally, the ADI score was dramatically decreased in the treatment group, with a remarkable 63.49% reduction, where the placebo group experienced only a 4.5% decrease. Park et al. [48] developed a complex system consisting of ceramide liposomes in a cellulose hydrogel, referred to as the liposome-in-hydrogel complex system. They compared various formulations and found that quercetin and rutin exhibited significantly higher skin permeability (quercetin, 67.42%; rutin, 59.82%) when delivered via the liposome-in-hydrogel complex system compared to single systems of hydrogel or liposome (quercetin, 31.77%; rutin, 26.35%) or the control (phosphate buffer, pH 7.4). This suggests that water-soluble antioxidants like quercetin and rutin can be more easily absorbed through the skin when delivered using a liposome-in-hydrogel system. In another study by Park et al. [49], they prepared a pH-responsive hydrogel for the transdermal delivery of naringenin. The hydrogel was based on carboxymethyl cellulose and 2-hydroxyethyl acrylate and was synthesized using radical polymerization. The hydrogel demonstrated a higher swelling ratio at pH 7.5 (the pH of acne-prone skin) and pH 8.5 (the pH of atopic skin) compared to pH-responsive hydrogel as a promising delivery method for topical treatment of atopic dermatitis.

### 6.4. Nanofibers

Nanofibers are one-dimensional nanomaterials with a diameter between tens and hundreds of nanometers, a high surface area-to-volume ratio, linked nanoporosity, fluid drainage, drug release rate, and better mass transport characteristics [50]. They have various applications, including biomedical, chemical, defense, and environmental protection. Biodegradable fibers are needed that are efficient against a variety of microbes, particularly those that are resistant to antibiotics, as microbial infections lead to prolonged and incomplete wound healing due to elevated inflammatory responses. A study was performed by Bartomiej et al. [51] on electrospun nanofibers containing quercetin. The quercetin nanofibers were reported to be 3.5 and 7 µm in diameter and to have a smooth and continuous appearance. Quercetin could not be released from PLA fibers after five days of incubation in PBS at 37 °C, indicating that it was firmly trapped within the fibers due to its poor solubility in water and the hydrophobic nature of polylactide. However, despite not being released into the environment, the quercetin retained its antibacterial activity, inhibiting the growth of bacteria in direct contact with the fibers. They found that the PLA nonwovens with quercetin were similarly effective at inhibiting the growth of *K. pneumoniae* and *E. coli* from the Enterobacteriaceae family. The nonwovens with quercetin were also more effective at inhibiting the growth of *S. aureus* than ofloxacin and clindamycin. Another study on a quercetin nanofiber membrane loaded with quercetin in Eudragit L-100 by Ao et al. [52] developed a quercetin-loaded Eudragit L-100 nanofiber membrane that exhibited high ductility and a controlled drug release rate. The addition of polyethylene glycol-4000 (PEG-4000) led to reduced fiber breakage and increased fiber length. Moreover, the crystallinity of the fiber membrane significantly decreased after incorporating PEG-4000. The testing indicated that the PEG-4000 modified fiber membrane had improved elongation at break. Additionally, in vitro drug release experiments showed rapid drug release at pH 7.4. The electospun Eudragit L-100 nanofiber membranes loaded with quercetin have promising applications in promoting the healing of skin, tissue, and joint injuries. Shababdoust et al. [47] used a similar electrospinning method to create nanofibers containing varying concentrations of curcumin in polyvinyl alcohol (PVA). Curcumin displays excellent antibacterial activity against gram-positive and gram-negative bacteria, making it suitable as an herbal antibacterial agent. Several PVA/C5 samples killed over 20% of gram-positive *S. aureus* bacteria after 6 h of contact with the bacteria medium. All organisms were destroyed within the first six hours of interaction. The cross-linked samples could kill over 50% of microbes in about 3 h. A study by Ali S. et al. [53] on the controlled release of curcumin-loaded nanofibers by electrospinning segmented polyurethanes shows steady curcumin release due to PEG chains in the soft segment. Antibacterial studies of the nanofibers loaded with curcumin against *E. coli* and *S. aureus* reported MICs of 163 μg·mL^−1^ and 175 μg·mL^−1^, respectively. Another study on PVA electrospun nanofibers containing Rhodomyrtus tomentosa was conducted by Senait S. et al. [54] Rhodomyrtus tomentosa extract consists of several phytochemicals with antioxidant, anti-inflammatory, antibacterial, antimalarial, antifungal, and osteogenic activities. Myricetin is the principal constituent found in the ethyl acetate extract of *R. tomentosa*. With its investigated antimicrobial activity against *E. faecalis*, *E. coli*, *P. aeruginosa*, and *B. subtilis*, this ethyl acetate extract is utilized to create nanofibers because it inhibits the growth of these microbes. The nanofiber diameter was found in a range of 120 nm to 214 nm in the field emission scanning electron microscopy (FE-SEM) study of Rhodomyrtus tomentosa extract (RTE) with 10% PVA. The antimicrobial activity of the electrospun RTE and PVA nanofibers against *E. faecalis*, *E. coli*, *P. aeruginosa*, and *B. subtilis* was investigated, and an inhibition zone ranging from 7 to 12 mm was observed. So, RTE/PVA electrospun nanofibers can be used as a bioactive agent for various bacterial infections. Krit S. et al. [55] developed propolis electrospun nanofibers with polylactic acid fibers (PLA). A homogeneous concentration of propolis in fibers was reported using a standard known concentration curve. Bactericidal studies of PLA/propolis nanofibers against *P. mirabilis*, *E. coli*, *S. epidermidis*, and *S. aureus* at a concentration of 2% (*w/v*) were reported. Therefore, based on the above findings, nanofibers could be a better option in the management of topical infectious disorders.

### 6.5. Inorganic Nanoparticles

Recent advancements in targeting specific cellular actions have shifted toward using inorganic nanoparticles, known for their stability over extended periods. Inorganic nanoparticles can be used to deliver drugs to cells because they are biocompatible, can be targeted, and can release drugs in a controlled way. These properties are helpful in cosmetics for anti-aging, anti-acne, and skin care products, as well as in treating skin diseases and transdermal delivery of substances. A standard method for producing metallic nanoparticles (MNPs) involves chemical reductants for reducing metal salts or complexes. These reductants include sugars, hydrazine, sodium citrate, sodium borohydride, sodium ascorbate, and sodium citrate. Flavonoids have been extensively used as reductants and capping agents in forming metallic nanoparticles. Metallic nanoparticles, typically composed of unreactive metals like silver, titanium, and gold, have also been extensively employed for controlled release. Madhurima P. et al. [56] used the methanolic seed extract of *P. pinnata* to make silver nanoparticles (AgNPs) in a green way. The phytochemicals in the extract served as capping and reducing agents. The AgNPs produced contained significant amounts of total flavonoids (88.87 ± 2.6%) and karanjin (95 ± 5.4%). The AgNPs were characterized using various techniques and found to have good antioxidant potential and antimicrobial activity against several bacteria. The AgNPs were incorporated into the gel. A zone of inhibition against *S. aureus* (25 ± 1.5 mm), *B. subtilis* (15 ± 0.6 mm), *P. aeruginosa* (24 ± 0.6 mm), and *E. coli* (20 ± 0.6 mm) was used to demonstrate the antibacterial activity of the AgNP-containing gel. The study also investigated the wound-healing potential of AgNPs by applying them topically to wounded rats, where they showed significant wound-healing activity compared to a commonly used betadine ointment. AgNPs have the potential for the treatment of topical infections and wound healing (as shown in Table 2). Another study of a similar nature was carried out by Jain et al. [57] to evaluate the function of biomolecules in the synthesis of AgNPs under various physicochemical conditions, employing quercetin, a derivative of Ocimum sanctum, as a precursor for the green synthesis of AgNPs. The study found that the leaf extract and quercetin separately produced AgNPs with similar characteristics and antibacterial activity against *E. coli* strains, indicating that the biomolecules present in Ocimum Sanctum, specifically quercetin, are responsible for reducing metal ions to form AgNPs. The zone of inhibition for both Ocimum sanctum extract-synthesized AgNPs and quercetin AgNPs was 14 mm. Muhammad Q.N. et al. [58] focused on a cost-effective and environmentally friendly method of synthesizing AgNPs using Ephedra procera C. A. Mey plant extract. Screening of plant aqueous extract showed significant total phenolic content and total flavonoid content values of 117 ± 0.78 µg/mg and 20.7 ± 0.21 µg/mg, respectively. It also exhibited good antioxidant properties, with a total antioxidant capacity and 2,2-dipheny-1-picrylhydrazyl scavenging potentials of 73.8 ± 0.32 µg/mg and 71.8 ± 0.73%, respectively. The green synthesized *E. procera* nanoparticles (EPNPs) effectively inhibit the growth of *E. coli* and *B. subtilis* with MICs of 11.12 µg/mL. They were also found to be effective against the fungal species *A. niger* and *A. flavus*. Milanezi et al. [59] synthesized quercetin-capped gold nanoparticles, where quercetin-capped gold nanoparticles (Qct-AuNPs) act as good antibacterial and antifungal agents. Their antioxidant properties were evaluated, as well as their antimicrobial and cytotoxic properties. The nanoparticles were small, spherical, smaller than 100 nm, and showed higher antioxidant activity than free quercetin. Metallic nanoparticles act as an active antimicrobial agent for various skin disease-causing pathogens. Moreover, they showed potent antifungal activity against Aspergillus fumigatus at concentrations between 0.1 to 0.5 mg/mL. The study suggests that Qct-AuNPs produced through a cost-effective method could have promising applications in various medical fields. Thus, the inorganic nanoparticles showed effective results against the various microorganisms mentioned above that cause skin infectious disease.

## 7. Challenges Associated with Nanoformulations

### 7.1. Challenges for Synthesis

It is fundamental to obtain the proper shape and size of the nanoparticles, but controlling the shape and size is yet to be achieved. The fabrication of nanoparticles with a narrow size distribution is often a challenging task due to the inherent tendency of flavonoids to aggregate or coalesce into larger particles [72]. Since nanoparticles using flavonoids are nowadays formulated with various classes of nanocarriers. Stabilization is a challenge since the susceptibility of flavonoids to degradation has been well documented under a range of conditions, including exposure to light, oxygen, and extreme pH levels. Optimization of the bio-efficacy and characteristics of the same type of nanoparticles synthesized using flavonoids via different synthesis methods is also a technical challenge for nanoparticle synthesis using flavonoids or natural compounds because there is a variation in their characteristics and bio-efficacy due to different synthesis techniques [73]. The direct incorporation of nanoparticles or hydrogels is limited due to the poor solubility of flavonoids in organic solvents and water, as they are natural compounds. Therefore, the encapsulation efficiency of nanocarriers can be influenced by various factors, including the solubility of flavonoids, compatibility with carrier materials, and formulation parameters. These factors collectively contribute to poor encapsulation.

### 7.2. Scale-Up Strategies and Current Good Manufacturing Practices (cGMPs) Processes

The compatibility of biomaterial-based hydrogels with cGMPs is a significant barrier to clinical translation and integration. Since hydrogels are prepared at a small scale in labs during the preclinical stage, it becomes necessary to make efforts to synthesize strategies for large-scale systems [74]. Large-scale production creates challenges such as batch variations, robustness, safety, and efficacy difficulties that become unavoidable. Employing natural polymers presents additional challenges due to their heterogeneity, potential variation in molecular-level properties, and the possibility of altered characteristics once they are synthesized, as in the case of hydrogel. Moreover, the high-water content of hydrogels adds complexity to the sterilization, storage, and production processes.

### 7.3. Challenges for Characterizations

Characterizing flavonoid nanoparticles and hydrogels presents several challenges due to their distinctive properties and intricate structures. The interactions between nanoparticles and target proteins and immune cells are influenced by various surface properties of the nanoparticles, including particle surface area, surface charge, chemical reactivity, and hydrophobicity [75]. These surface properties play a crucial role in determining the nature and extent of these interactions. Henceforth, it is crucial to subject these parameters to critical analysis. However, there is currently a lack of standardized methodologies for evaluating hydrophobicity, quantifying and assessing the homogeneity of surface functionalities, and determining the surface area, making it a great challenge for scientists [76]. Additionally, analyzing the drug loading and release potential of nanoparticles and hydrogels is a notable challenge. Though there are some techniques to determine the drug loading and drug-release profile from nano-formulations, such as liquid chromatography coupled with mass spectroscopy (LC-MS/MS) and inductively coupled mass spectroscopy (ICP-MS) [77]. These technologies are still incompetent to quantify the active ingredient with associated impurities therein because they are substance-specific and are emerging as a challenge to quantify and determine the recently developed active pharmaceutical ingredients such as DNA, mRNA, and nucleic acid. Moreover, the characterization of the release profile of encapsulated flavonoids from carriers showcases a significant challenge owing to the intricate nature of the release mechanism and the interplay between the drug, flavonoid matrix, and the surrounding environment. Another challenge is analyzing the encapsulation potential of flavonoid nanoparticles because flavonoids and the carrier matrix could interact, flavonoids could leak out, or the encapsulation could be incomplete.

### 7.4. Technology Challenges

Despite the notable achievements achieved by hydrogel-based delivery systems, significant technological hurdles impede their effective clinical implementation. These problems encompass several aspects, such as chemistry, production and controls, regulatory standards, and practical adaptation. Due to the intricate nature of hydrogel manufacture and the inherent variability across different hydrogel systems, the expenses associated with developing hydrogel technology through clinical translation have been estimated to range from $50 million to $800 million [74].

### 7.5. Regulatory Approvals

The regulatory categorization and licensing of hydrogel scaffolds are complex due to the wide range of cross-linking agents and biomaterials used in their development. In contrast to pharmaceuticals, which are categorized in a wide manner, hydrogels are classified within the “devices” category as defined by Section 201(g) of the Federal Food, Drug, and Cosmetic (FD&C) Act as “any product which does not archive its primary intended purpose through chemical action within or on the body.” In addition, except for a few cases, most hydrogel-based products must undergo an extra assessment by the Food and Drug Administration (FDA) by submitting a 510(k) Pre-Market Notification to acquire legal marketing authorization in the USA [77]. Hydrogel scaffolds containing drugs or drug-secreting cells are classified as combination products, requiring a regulatory clearance process that typically spans 7–10 years. This extended timeframe significantly constrains their economic feasibility.

### 7.6. Other Clinical Challenges

Despite the achievements attained in the hydrogel-based delivery system, several challenges persist in hydrogel drug delivery. These challenges encompass the occurrence of burst release upon administration, the limited capacity to encapsulate specific drug categories (e.g., hydrophobic drugs, proteins, antibiotics, and nucleic acids), and the restricted ability to finely adjust geometric patterns and shapes for precise control of drug release [78]. The current focus of clinical trials primarily revolves around evaluating hydrogel dressings in the post-market phase. Results from these trials suggest that hydrogels improve the appearance of healed burn wounds compared to standard care [78]. The observed enhancements include reduced pain during dressing changes, improved wound re-epithelization, and decreased necessity for surgical excision or grading [79]. Despite these benefits, no hydrogel has yet demonstrated efficacy in promoting burn wound regeneration or effectively combating infection. The hydrophilic nature of hydrogels also presents challenges in displacing water from the adhesive interface. As a result, ongoing research emphasizes the development of more compatible, effective, and stable interfaces to advance hydrogel-based wound dressings.

## 8. Conclusions

Flavonoids have been investigated for their antimicrobial effects in recent decades. However, their poor aqueous solubility limits their oral bioavailability, and they are quickly degraded and metabolized in the body, making oral delivery challenging. So, nanoformulations and delivery methods, like cutaneous administration, have been made to make flavonoids more soluble and allow them to pass through the skin barrier with fewer side effects. Flavonoid encapsulation into nanoformulations is an effective way to improve their pharmacokinetics and safety. However, obstacles like burst release and skin retention time must be solved to reach acceptable therapeutic doses. The nano-formulations of natural products proven to have magnificent biological potency will be a remarkable pharmacophore for several challenging topical infections, whereas the challenges facing the development of nanoformulations on a large scale to commercialize them require fixing those issues in the near future.

## Figures and Tables

**Table 2:** Examples of flavonoid nanoformulations that alleviate topical infections.

Type of Nano-Formulation	Flavonoid/Source of Flavonoid Used (Extract Used)	Method of Preparation	Size	Targeted Topical Infection	References
Nanofiber	Quercetin	Electrospinning	550 ± 113	*Candida albicans*	[60]
	Propolis	Electrospinning	419 nm	*S. aureus*, *Staphylococcus epidermidis* and *Candida albicans*	[61]
	Propolis	Electrospinning	150–400 nm	*S. aureus*, *S. epidermidis*, *Proteus mirabilis* and *E. coli*	[55]
	*Camomilla reticutita* (L)	D-optimal design	175 nm	*S. aureus*, *Candida albicans*	[62]
	Curcumin	Electrospinning	113 ± 31 nm	*S. aureus*, *E. coli*	[63]
Hydrogel	*Mangifera indica* leaf extract	Dispersion method	-	*S. aureus*	[64]
	*Borassus flabellifer* L.	Dispersion method	-	*Cutibacterium acnes*	[65]
	*Nigella sativa* L.	Dispersion method	-	*Acne vulgaris*	[47]
Liposomes	(+)-catechin	Reverse-phase evaporation	551.1 ± 53.4	*P. aeruginosa*, *E. coli*	[32]
	Propolis	Modified ethanol injection method	450.8 ± 40.87 nm	*S. aureus*, *E. faecalis*, *P. aeruginosa*, *E. coli*	[66]
	Epigallocatechin gallate	Extrusion method	93.2 ± 80.22 nm	Wound infection by *S. aureus*	[67]
	Curcumin	-	147 ± 6 nm	*S. aureus*, *S. epidermidis*, *Enterococcus faecalis*, *Bacillus cereus*, *B. subtilis*	[5]
Zinc oxide nanoparticles	*Salvia officinalis* L.	Green synthesis	26.14 nm	*C. albicans*	[68]
Silver nanoparticle	*Pongamia pinnata*	Green synthesis	20 to 60 nm	*S aureus*, *Escherichia coli*, *Bacillus subtilis* And *Pseudomonas aeruginosa*	[56]
	*Pouzolzia zeylanica*	Green synthesis	5–49 nm	*A. niger*, *A. flavus*, and *F. oxysporum*	[69]
Gold nanoparticles	*Salix alba* L.	Green synthesis	50–80 nm	*S. aureus*, *A. solani* and *A. niger*	[70]
Polymeric nanoparticle	Curcumin	Desolvation method	110 nm	*P. aeruginosa*, *S. aureus*	[41]
	Quercetin	Water/oil/water (w/o/w) emulsion–solvent evaporation method	100–150 nm	*E. coli*, *M. tetragenus*	[42]
	Propolis	Ionic gelation method with modification	247.1 nm to 512.3 nm	*Enterococcus faecalis*	[71]

## Data Availability

The authors confirm that the data supporting the findings of this study are available within the articles and can be shared upon request.
